# Bidirectional Association Between Tuberculosis and Chronic Obstructive Pulmonary Disease: A Systematic Review and Meta-Analysis

**DOI:** 10.3390/jcm14217639

**Published:** 2025-10-28

**Authors:** Jingyuan Feng, Minghao Hu, Hongfei Duan

**Affiliations:** Department of Tuberculosis, Beijing Chest Hospital, Capital Medical University, Beijing 101125, China; 112025011297@mail.ccmu.edu.cn (J.F.); minghaohu@mail.ccmu.edu.cn (M.H.)

**Keywords:** Tuberculosis, COPD, Risk factors, Bidirectional association

## Abstract

**Background:** Tuberculosis (TB) and chronic obstructive pulmonary disease (COPD) are major respiratory diseases contributing to high global morbidity and mortality. Recent studies suggest a potential bidirectional association between them; however, the overall evidence has not been systematically integrated. This study aims to comprehensively evaluate the bidirectional epidemiological association between TB and COPD through a systematic review and meta-analysis. **Methods:** We systematically searched observational studies published from database inception to 31 August 2025, in PubMed, Embase, Web of Science, and other databases. Data were extracted from studies examining the risk of COPD development in individuals with a history of TB and the risk of TB development in COPD patients. Pooled effect sizes were calculated using random-effects models, including pooled odds ratios (ORs) and prevalence rates, with assessments of heterogeneity and publication bias. **Results:** A total of 32 studies were included, involving over 670,000 participants. Meta-analysis revealed that individuals with a history of TB had a significantly increased risk of developing COPD (pooled OR = 2.46, 95% CI: 1.95–3.10). Similarly, COPD patients had a significantly elevated risk of developing TB (pooled OR = 2.21, 95% CI: 1.57–3.11). The pooled prevalence of COPD among TB patients was 15.95% (95% CI: 11.61–21.53), while the pooled prevalence of TB among COPD patients was 5.57% (95% CI: 2.24–13.18). Significant heterogeneity was observed, but no substantial publication bias was detected. **Conclusions:** A significant and robust bidirectional association exists between TB and COPD, with each being an important independent risk factor for the other. These findings underscore the necessity of integrated screening and comorbidity management for both diseases in clinical practice and public health strategies, particularly in high TB burden regions. Prospective studies are warranted to further elucidate causal mechanisms and evaluate interventions.

## 1. Background

Tuberculosis (TB), caused by *Mycobacterium tuberculosis*, remains a major global public health challenge. According to the World Health Organization, there were approximately 10.6 million new TB cases and 1.3 million deaths worldwide in 2022, making TB the leading cause of death from a single infectious agent [1,2]. The burden is particularly heavy in low- and middle-income countries (LMICs), among people living with HIV, and in regions with limited healthcare resources. TB can lead to pulmonary cavitation, fibrosis, irreversible lung damage, and dissemination to organs such as the brain and spine, resulting in high disability and mortality rates. The spread of drug-resistant TB (e.g., MDR-TB and XDR-TB) further complicates treatment, increases costs, and reduces cure rates. Additionally, TB often coexists with chronic conditions like COPD and diabetes, increasing disease complexity and management challenges. The long-term nature of TB, loss of productivity, and social stigma often plunge affected households into poverty, exacerbating socioeconomic burdens [3].

Meanwhile, chronic obstructive pulmonary disease (COPD) is a heterogeneous disease characterized by persistent airflow limitation, involving chronic airway inflammation, emphysema, and airway remodeling [4]. COPD imposes a substantial global burden, causing approximately 3.23 million deaths in 2019, ranking as the third leading cause of death worldwide [5,6]. Although smoking is the primary risk factor, growing evidence indicates that non-smoking-related risk factors—such as air pollution, occupational exposures, and previous infections, particularly tuberculosis—play significant roles in COPD development in LMICs [7,8,9]. Notably, over 90% of COPD-related morbidity and mortality occur in LMICs, where a considerable proportion of patients have no smoking history, suggesting that other factors (e.g., post-infection lung injury) may be important in COPD pathogenesis.

Both TB and COPD are major drivers of global respiratory disease burden, particularly in high TB burden LMICs, presenting overlapping public health challenges. The association between COPD and TB has been debated, with studies reporting both positive and null associations. Recent large-scale epidemiological and genetic studies, including Mendelian randomization analyses, indicate that a history of TB significantly increases the risk of COPD, independent of traditional risk factors like smoking [10,11]. Conversely, evidence for COPD increasing TB risk is relatively weaker and may be influenced by confounders such as corticosteroid use and socioeconomic status [12,13]. Some studies highlight overlapping inflammatory and immune pathways, suggesting that TB-induced lung damage and persistent inflammation can lead to airflow limitation and a distinct TB-associated COPD phenotype [14,15]. However, other studies report no significant impact of TB history on COPD mortality or exacerbation severity [11,14,16]. Due to methodological limitations and potential confounding, the causal direction remains controversial.

In this context, this study aims to comprehensively elucidate the bidirectional association between TB and COPD through a systematic review and meta-analysis. We evaluate the risk and effect size of COPD development in individuals with a history of TB and quantify the association strength of TB prevalence in COPD patients. By synthesizing global evidence, we seek to clarify the mutual influences on epidemiological patterns, clinical features, and prognostic differences. The synthesis of evidence from 32 studies enhances the statistical power to establish this association and underscores its public health significance. These results highlight the potential for integrated screening and comorbidity management strategies in clinical practice, particularly in high TB burden regions. Furthermore, our study lays a foundational epidemiological basis for future research aimed at elucidating the underlying mechanisms and developing precision interventions for this comorbid condition.

## 2. Methods

### 2.1. Search Strategy

We systematically searched the following electronic databases: PubMed, Embase, Web of Science Core Collection, and Cochrane Library. The search period extended from database inception to 31 August 2025. The search strategy combined subject headings (MeSH, Emtree) and free-text terms, adapted to the syntax rules of each database. Core search terms included: “Tuberculosis,” “Pulmonary Tuberculosis,” “TB,” “Chronic Obstructive Pulmonary Disease,” “COPD,” “Emphysema,” “Chronic Bronchitis,” “prevalence,” “prevalence,” “risk,” “cohort study,” “case–control study,” “cross-sectional study,” etc. The PubMed search strategy is provided as an example: (“Tuberculosis” [Mesh] OR “Tuberculosis, Pulmonary” [Mesh] OR “TB” [tiab] OR “pulmonary TB” [tiab]) AND (“Pulmonary Disease, Chronic Obstructive” [Mesh] OR “COPD” [tiab] OR “COAD” [tiab] OR “emphysema” [tiab] OR “chronic bronchitis” [tiab]) AND (“prevalence” [tiab] OR “prevalence” [tiab] OR “risk” [tiab] OR “odds ratio” [tiab] OR “cohort studies” [Mesh] OR “case–control studies” [Mesh] OR “cross-sectional studies” [Mesh]).

Additionally, we manually screened reference lists of included studies to identify additional relevant publications.

### 2.2. Inclusion and Exclusion Criteria

Inclusion criteria were as follows: observational studies (prospective or retrospective cohort, case–control, or cross-sectional designs) published in English. Studies were categorized based on association direction: (1) For TB’s effect on COPD risk, the exposed group consisted of individuals with a history of active or cured pulmonary TB, compared to those without; (2) For COPD’s effect on TB risk, the exposed group comprised individuals meeting COPD diagnostic criteria, compared to non-COPD controls. Participants were not restricted by gender, ethnicity, nationality, or smoking status. For Direction 1, TB exposure required bacteriological (e.g., sputum smear/culture), molecular (e.g., GeneXpert), or clinical confirmation. For Direction 2, COPD exposure required spirometric confirmation (post-bronchodilator FEV_1_/FVC < 0.70) or clinical diagnosis (e.g., GOLD guidelines). Control groups had no history of the respective exposure. Primary outcomes included: for Direction 1, incident or prevalent COPD (spirometrically or clinically confirmed); for Direction 2, incident active TB (reactivation or reinfection, bacteriologically/molecularly or clinically confirmed). Secondary outcomes included studies providing risk estimates (OR with 95% CI).

Exclusion criteria: reviews, meta-analyses, case reports, conference abstracts, commentaries; studies lacking extractable effect measures (e.g., OR and 95% CI); animal or pediatric studies (age < 18 years); non-English publications.

### 2.3. Study Selection and Data Extraction

Two reviewers independently screened titles and abstracts, assessed full texts, and extracted data using a predefined form. Discrepancies were resolved through discussion or arbitration by a third reviewer. Extracted data included: first author, publication year, region, study design, sample source, follow-up duration (for cohorts), participant characteristics (age, gender, smoking history, etc.), exposure/outcome definitions, sample size, effect estimates (OR and 95% CI), and adjusted confounders. Corresponding authors were contacted for missing data when necessary.

### 2.4. Quality Assessment

Two reviewers independently assessed the quality of cohort and case–control studies using the Newcastle-Ottawa Scale (NOS) [17]. The NOS evaluates selection (max 4 stars), comparability (max 2 stars), and outcome/exposure (max 3 stars), with a total score of 9. Studies scoring ≥7, 5–6, and <5 were considered high, moderate, and low quality, respectively.

### 2.5. Statistical Analysis

All meta-analyses were conducted using R software (version 4.3.0). We performed two sets of analyses: (1) pooling odds ratios (ORs) with 95% confidence intervals (CIs) from observational studies to evaluate bidirectional associations, and (2) pooling prevalence or prevalence rates with 95% CIs to quantify disease burden. For studies providing raw data, we calculated ORs from 2 × 2 tables or rates from event counts. An OR quantifies association strength; for example, an OR of 2.0 indicates the odds of the outcome are twice as high in the exposed versus control group. Given expected heterogeneity, we used random-effects models for all pooled estimates. Heterogeneity was assessed using Cochran’s Q test (*p* < 0.10) and the I^2^ statistic (I^2^ > 50% and >75% indicating moderate and substantial heterogeneity, respectively). Publication bias was assessed using funnel plots and Egger’s regression test. Funnel plot asymmetry, or a significant Egger’s test (*p* < 0.05), suggests potential bias. Sensitivity analyses were performed by sequentially excluding each study to test result robustness. All *p*-values were two-tailed, with *p* < 0.05 considered statistically significant.

### 2.6. Reporting and Registration

This systematic review and meta-analysis was conducted in accordance with the Preferred Reporting Items for Systematic Reviews and Meta-Analyses (PRISMA) guidelines. The completed PRISMA checklist is provided as Table S1. The review protocol was not registered.

## 3. Results

### 3.1. Literature Search and Selection

**Figure 1:**
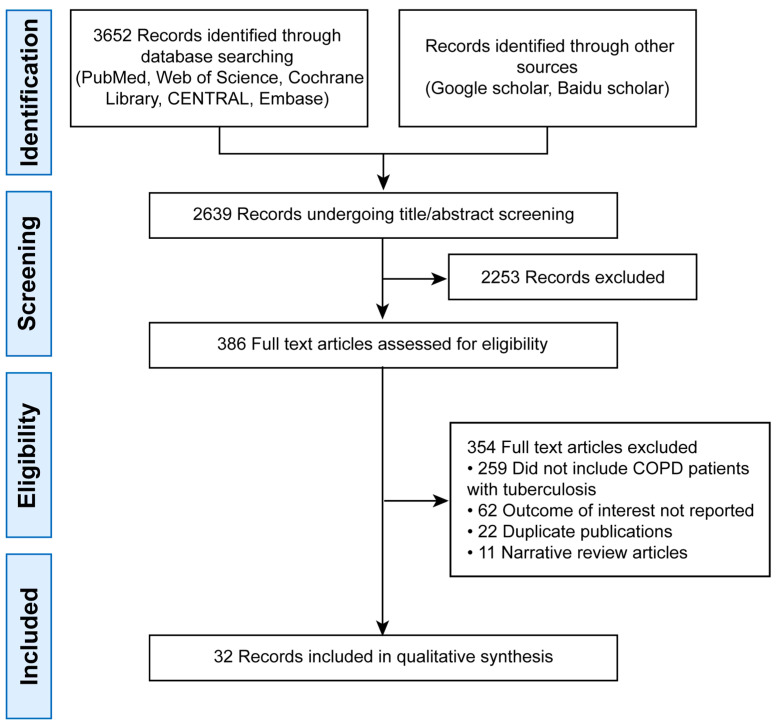
PRISMA Flow Diagram of Study Selection.

The initial search yielded 3652 records. After deduplication, 2639 remained. Title/abstract screening excluded 2253 articles, leaving 386 for full-text review. Finally, 32 studies were included (Figure 1).

### 3.2. Characteristics and Quality of Included Studies

The 32 included observational studies, published between 2007 and 2025, were conducted across Asia (e.g., China, South Korea, India), Africa, North America, and Europe. Among them, two adopted a case–control design and 30 were cross-sectional studies.

For the outcome “Risk of TB in COPD Patients,” 10 studies were included, while 13 studies contributed to the analysis of “Risk of COPD in TB Patients.” Additionally, 15 studies reported on the “Prevalence of COPD in TB Patients,” and 20 studies provided data on the “Prevalence of TB in COPD Patients.”

The NOS score across studies was above 6 (out of 9), indicating generally good methodological quality. Detailed study characteristics are summarized in Table 1.

**Table 1:** Characteristics and Quality of Included Studies.

Author	Year	Location	Study Design	Age (Years)	Definition of Tuberculosis	COPD Definitions	NOS
Menezes [18]	2007	Latin America	cross-sectional	56.6 ± 11.9	TB history (self-reported)	Post-BD FEV1/FVC < 70%	6
Caballero [19]	2008	Colombia	cross-sectional	55.8 ± 11.2	TB history (self-reported)	Post-BD FEV1/FVC < 70%	7
Lam [20]	2010	China	cross-sectional	61.9 ± 6.9	TB history (self-reported) or TB lesions on CXR	Pre-BD FEV1/FVC < LLN	8
Inghammar [21]	2010	Sweden	cross-sectional	NA	TB history (self-reported)	Post-BD FEV1/FVC < 70% or FEV1/FVC < LLN	8
Lamprecht [22]	2011	Austria	cross-sectional	57	TB history (self-reported)	Pre-BD FEV1/FVC < 70% or FEV1/FVC < LLN	6
Idolor [23]	2011	Philippines	cross-sectional	52.9 ± 9.9	TB history (self-reported)	Post-BD FEV1/FVC < 70%	7
Lee [24]	2011	Korea	cross-sectional	43.1	TB lesions on CXR	Pre-BD FEV1/FVC < 70% or FEV1/FVC < LLN	7
Govender [25]	2011	South Africa	case–control	NA	TB history (self-reported) and confirmed by doctor	Pulmonologist diagnosis	8
Lee [26]	2012	Taiwan	cross-sectional	54.5	Medical history	Medical history	8
Danielsson [27]	2012	Sweden	cross-sectional	58.8	TB history (self-reported)	Pre-BD FEV1/FVC < 70% or FEV1/FVC < LLN	8
Hagstad [28]	2012	Sweden	cross-sectional	NA	TB history (self-reported)	Post-BD FEV1/FVC < 70%	8
Jo [29]	2014	Korea	cross-sectional	53.55	Medical history	Medical history	7
Gemert [30]	2015	Uganda	cross-sectional	45	TB history (self-reported)	Post-BD FEV1/FVC < LLN	8
Jung [31]	2015	Korean	cross-sectional	57.1 ± 10.9	Medical history	FEV1/FVC% < 70	8
Zhao [32]	2015	China	cross-sectional	64.64	Medical history	Post-BD FEV1/FVC < 70%	7
Yang [33]	2015	China	case–control	NA	Medical history	Post-BD FEV1/FVC < 70%	8
Viet [34]	2015	Vietnam	cross-sectional	53.9 ± 11.6	TB history (self-reported)/TB lesions on CXR	Pre-BD FEV1/FVC < 70%	6
Choi [35]	2017	Korea	cross-sectional	55.6 ± 1.6	TB history (self-reported)	Post-BD FEV1/FVC < 70% or FEV1/FVC < LLN	7
Sobrino [36]	2017	Argentina	cross-sectional	NA	TB history (self-reported)	Post-BD FEV1/FVC < 70% or FEV1/FVC < LLN	6
Magitta [37]	2017	Tanzania	cross-sectional	51.8 ± 10.6	Medical history	Post-BD FEV1/FVC < 70%	6
Nishida [38]	2017	Japan	cross-sectional	58	TB history (self-reported)	Post-BD FEV1/FVC < 70%	6
Wang [39]	2018	China	cross-sectional	43.8 ± 0.8	TB history (self-reported)	Post-BD FEV1/FVC < 70%	7
Nugmanova [40]	2018	Ukraine, Kazakhstan and Azerbaijan	cross-sectional	41.29	TB history (self-reported)	Post-BD FEV1/FVC < LLN	6
Gupte [41]	2019	India	cross-sectional	32	TB history (self-reported)	Post-BD FEV1/FVC < 70%	6
Bekele [42]	2020	Ethiopia	cross-sectional	42.7	TB history (self-reported)	Post-BD FEV1/FVC < 70% or FEV1/FVC < LLN	7
Guo [43]	2020	China	cross-sectional	38	TB history (self-reported)	Pre-BD FEV1/FVC < 70%	7
Balan [44]	2022	India	cross-sectional	NA	TB history (self-reported)	Pre-BD FEV1/FVC < 70%	6
Mohamed A [45]	2022	Egypt	cross-sectional	52.77	TB history	Post-BD FEV1/FVC < 70% or FEV1/FVC < LLN	8
Massongo [46]	2023	Cameroon	cross-sectional	43	Medical history	Medical history	8
Wang [47]	2023	China	cross-sectional	52.8 ± 9.4	Medical history	Pre-BD FEV1/FVC < 70%	8
Kim [48]	2024	Korea	cross-sectional	57.2 ± 11.2	Definition of tuberculosis	COPD definitions	8
Zeng [49]	2024	China	cross-sectional	57.91	TB history (self-reported)	Post-BD FEV1/FVC < 70%	6

Note: COPD, chronic obstructive pulmonary disease; TB, tuberculosis; CXR, chest X-ray; FEV1, forced expiratory volume in 1 *s*; FVC, forced vital capacity; LLN, lower limit of normal; NA, not available.

### 3.3. Risk of COPD in TB Patients

**Figure 2:**
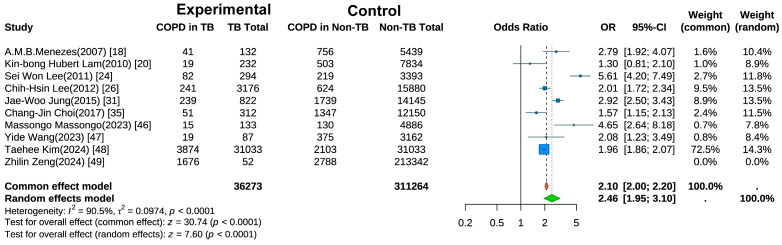
Forest Plot of Meta-Analysis on the Association of Tuberculosis with COPD Risk.

Ten studies examined the risk of COPD development in individuals with a history of TB [18,20,24,25,26,31,35,47,48,49]. A random-effects meta-analysis (Figure 2) revealed a significantly elevated risk of COPD in the TB group compared with controls (pooled OR = 2.46, 95% CI: 1.95–3.10, *p* < 0.001). Substantial heterogeneity was observed (I^2^ = 90.5%, *p* < 0.001), suggesting variations across studies in terms of population characteristics, study designs, or outcome measurements. Sensitivity analysis (Figure S1) using the leave-one-out method showed that after excluding the study by Kim et al. [48], the heterogeneity decreased to I^2^ = 88.9%.

### 3.4. Risk of TB in COPD Patients

**Figure 3:**
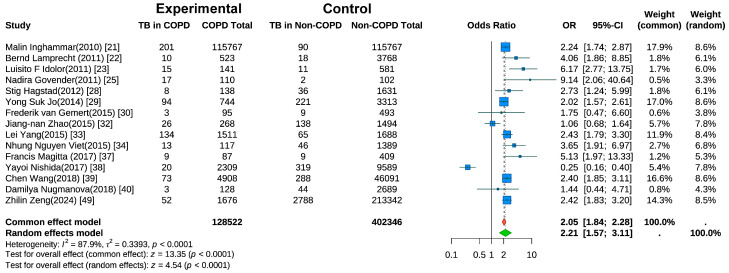
Forest Plot of Meta-Analysis on the Association of COPD and Risk of Tuberculosis.

Fifteen studies evaluated the risk of active TB in COPD patients [21,22,23,25,28,29,30,32,33,34,37,38,39,40,49]. Random-effects meta-analysis (Figure 3) revealed a significantly higher TB risk in COPD patients than in non-COPD controls (pooled OR = 2.21, 95% CI: 1.57–3.11, *p* < 0.001). Substantial heterogeneity was present (I^2^ = 87.9%, *p* < 0.001). A sensitivity analysis (Figure S2) using the leave-one-out method indicated that the heterogeneity was substantially reduced to I^2^ = 56.8% after the exclusion of the study by Nishida et al. [38].

### 3.5. Prevalence of COPD in TB Patients

**Figure 4:**
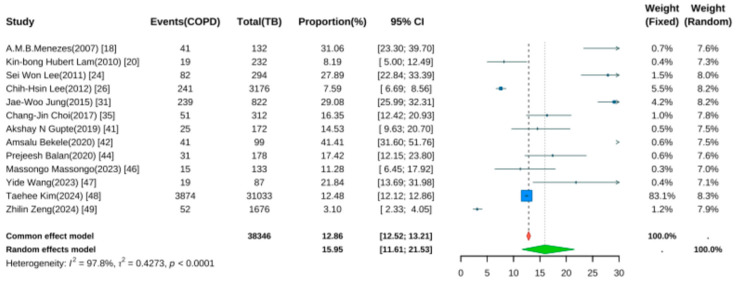
Forest Plot for the Prevalence of COPD in Pulmonary Tuberculosis Patients.

Thirteen studies reported the prevalence of COPD following TB [18,20,24,25,26,31,35,41,42,44,47,48,49]. Pooled analysis using a random-effects model (Figure 4) yielded a prevalence rate of 15.95% (95% CI: 11.61–21.53, *p* < 0.001), indicating that approximately one in six TB patients developed COPD during follow-up. Significant heterogeneity was noted (I^2^ = 97.8%, *p* < 0.001).

### 3.6. Prevalence of TB in COPD Patients

**Figure 5:**
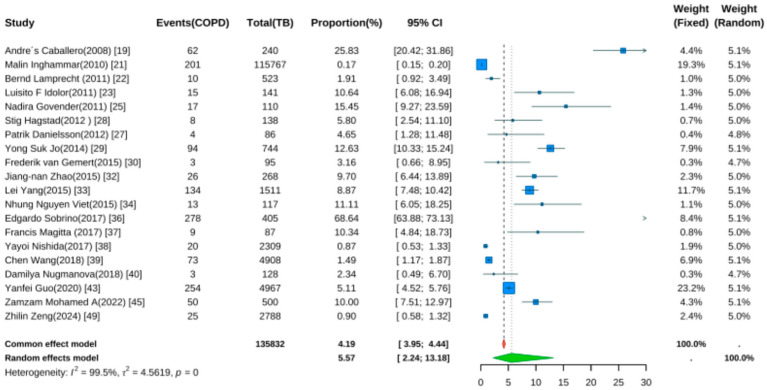
Forest Plot for the Prevalence of Active Tuberculosis in COPD Patients.

Twenty studies reported TB prevalence in COPD patients [19,21,22,23,25,27,28,29,30,32,33,34,36,37,38,39,40,43,45,49]. Pooled analysis (Figure 5) showed a prevalence rate of 5.57% (95% CI: 2.24–13.18, *p* < 0.001). Despite wide confidence intervals, the result was statistically significant, indicating elevated TB risk in COPD patients. Significant heterogeneity was noted (I^2^ = 99.5%, *p* < 0.001).

### 3.7. Publication Bias Assessment

**Figure 6:**
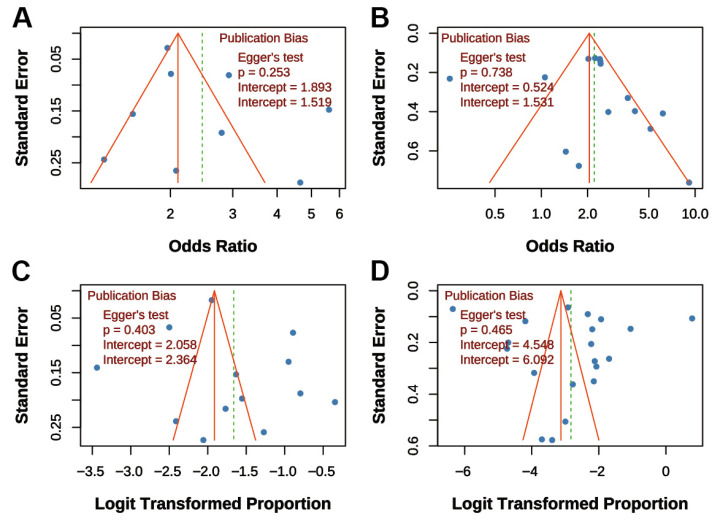
Funnel Plots for Publication Bias Assessment. (**A**) Association between TB and COPD risk; (**B**) Association between COPD and TB risk; (**C**) Prevalence of COPD post-TB; (**D**) Prevalence of TB in COPD patients. Dots represent individual studies. All funnel plots show approximate symmetry, and Egger’s tests indicated no significant publication bias (all *p* > 0.05).

Funnel plots and Egger’s tests were performed for all four main analyses. For “TB on COPD risk,” the funnel plot was symmetrical, and Egger’s test was non-significant (*p* = 0.253; Figure 6A). Similarly, for “COPD on TB risk,” the funnel plot showed symmetry, and Egger’s test was non-significant (*p* = 0.738; Figure 6B). For “COPD prevalence post-TB,” the funnel plot was symmetrical, and Egger’s test was non-significant (*p* = 0.403; Figure 6C). For “TB prevalence in COPD,” the funnel plot was symmetrical, and Egger’s test was non-significant (*p* = 0.465; Figure 6D). Overall, funnel plots displayed approximate symmetry, and Egger’s tests indicated no significant publication bias (all *p* > 0.05), supporting the reliability of the findings.

## 4. Discussion

This meta-analysis provides robust quantitative evidence confirming a significant bidirectional association between tuberculosis (TB) and chronic obstructive pulmonary disease (COPD). Our principal findings indicate that a history of TB is associated with a 2.46-fold increased risk of developing COPD, while pre-existing COPD is associated with a 2.21-fold elevated risk of incident active TB. Pooled prevalence data further substantiate this substantial disease burden, revealing that approximately 16% of TB patients developed COPD during follow-up, whereas nearly 6% of COPD patients developed TB. These results, which were statistically significant and consistent across analyses, underscore a strong epidemiological link between the two conditions.

Our findings are consistent with and reinforce those of prior studies. For instance, Fan et al. reported a significantly elevated risk of COPD among individuals with a history of TB (pooled OR = 2.59, 95% CI: 2.12–3.15) [50]. Another systematic review found a pooled odds ratio of 3.05 (95% CI: 2.42–3.85) for the association between prior TB and COPD in adults over 40 years of age, with the strongest associations observed in TB-endemic regions and among never-smokers [51]. Furthermore, pooled analyses of population-based studies conducted in low-resource settings indicated that COPD was approximately four times more prevalent among individuals with previous TB (25.7% vs. 8.3%), yielding an adjusted odds ratio of 3.78 (95% CI: 2.87–4.98) [52].

While our meta-analysis revealed substantial statistical heterogeneity, a common feature in syntheses of observational studies, this does not invalidate the primary findings. Instead, it reflects the real-world diversity of the included studies in terms of populations, diagnostic criteria for both TB and COPD, and study designs. Our qualitative systematic review of the included literature identified that variations in the definition of TB exposure (e.g., self-report versus microbiological confirmation) and COPD outcome (e.g., spirometric versus clinical diagnosis) were key contributors to this heterogeneity. Importantly, despite these methodological differences, the effect estimates from the vast majority of studies consistently pointed towards an increased risk, and no substantial publication bias was detected. This consistency in the direction of effect, coupled with the biological plausibility of the association, greatly enhances the credibility of our conclusions.

From a pathophysiological perspective, the bidirectional association we observed has a multilayered biological basis. TB-induced lung damage extends far beyond the acute infection phase, potentially initiating a long-term pathological process involving persistent immune dysregulation, MMP-mediated extracellular matrix destruction, and impaired tissue repair, ultimately leading to irreversible airflow limitation [53]. TB can cause persistent airway obstruction through direct tissue damage and secondary repair responses, including extensive fibrosis and airway wall remodeling. Even after microbiological cure, TB can leave structural sequelae such as bronchiectasis and emphysema, gradually evolving into a distinct COPD phenotype [54]. Conversely, COPD patients exhibit innate immune dysfunction, including impaired alveolar macrophage phagocytosis and disrupted airway mucosal barriers, creating conditions favorable for *Mycobacterium tuberculosis* colonization and reactivation [16,53,54,55,56]. Furthermore, frequent COPD exacerbations and long-term inhaled corticosteroid therapy may further suppress local immune surveillance, increasing the risk of latent infection reactivation [26,57]. At the molecular level, the comorbid state is characterized by persistent CD4^+^ T cell activation and heightened neutrophilic inflammation, forming a vicious cycle of “infection–inflammation–tissue destruction [53].

These mechanisms are supported by clinical observations that TB-COPD comorbidity presents unique phenotypic features, often identified on imaging as up-per-lobe-predominant fibrotic streaks and traction bronchiectasis. The recognition of this robust bidirectional association represents a critically important public health challenge, particularly in LMICs with high TB burden. Our findings therefore underscore the necessity of moving beyond viewing these as independent entities and towards integrated management strategies. In clinical practice, this implies considering long-term lung function monitoring for TB survivors and maintaining a high index of suspicion for active TB in COPD patients, especially those on corticosteroid therapy. For public health policy, this calls for the integration of TB and COPD screening and management programs to alleviate the dual burden of comorbidity.

Several limitations warrant cautious interpretation. First, all included studies were observational; although they demonstrated a significant positive association, causality cannot be established. While TB-induced structural damage supports biological plausibility, reverse causation (e.g., COPD patients having increased TB reactivation risk due to immunosuppression or steroid use) may also exist [50,56,58]. Bidirectional mechanisms require validation in prospective studies. Second, significant heterogeneity may stem from variations in TB exposure definitions (often self-reported, prone to recall bias) or residual confounding (e.g., inadequate adjustment for indoor air pollution). However, all effect estimates consistently showed positive associations, and sensitivity and subgroup analyses (e.g., radiologically confirmed TB) yielded robust results, enhancing credibility. Third, some studies relied on medical coding for outcome/exposure definitions, potentially introducing misclassification bias. The lack of stratified analyses based on TB severity, treatment details, and COPD phenotypes limited exploration of effect modifiers [59].

## 5. Conclusions

This meta-analysis provides conclusive evidence for a significant bidirectional association between TB and COPD. These findings underscore the vicious cycle between these two major respiratory diseases and call for integrated management strategies. For clinical translation, we recommend the following: (1) implementing routine TB symptom screening and low-threshold diagnostic testing in COPD management protocols, particularly in high-burden settings; (2) establishing post-TB spirometry and long-term respiratory monitoring for TB survivors to enable early COPD detection. These straightforward yet crucial steps can bridge the gap between epidemiological evidence and clinical practice, ultimately improving outcomes for patients affected by this comorbid condition. Future studies should focus on validating the cost-effectiveness of these integrated approaches and elucidating the underlying mechanisms to inform targeted interventions.

## Data Availability

The raw data supporting the conclusions of this article will be made available by the authors on request.

## Supplementary Materials

The following supporting information can be downloaded at: https://www.mdpi.com/article/10.3390/jcm14217639/s1, Table S1. PRISMA checklist; Figure S1. Sensitivity analysis for the association between tuberculosis and COPD risk using the leave-one-out method; Figure S2. Sensitivity analysis for the association between COPD and tuberculosis risk using the leave-one-out method.
