# Supplementary material for: Bidirectional Association Between Tuberculosis and Chronic Obstructive Pulmonary Disease: A Systematic Review and Meta-Analysis
Source: J Clin Med. 2025 Oct 28;14(21):7639. doi: 10.3390/jcm14217639 (PMC12608156; doi:10.3390/jcm14217639)
Supplement: Supplementary file 1 [file jcm-14-07639-s001.zip › jcm-3881939-supplementary-Figures S1 amd S2.pdf]

Bidirectional Association Between Tuberculosis and Chronic Obstructive Pulmonary Disease: A Systematic Review and Meta-Analysis

Jingyuan Feng, Minghao Hu and Hongfei Duan *

1. Department of Tuberculosis, Beijing Chest Hospital, Capital Medical University, Beijing, China

∗ Corresponding author. Hongfei Duan, Beiguan St, No. 9, Tongzhou Qu, Beijing 101149, People's Republic of China. E-mail address: duanhongfei@hotmail.com (H. Duan).

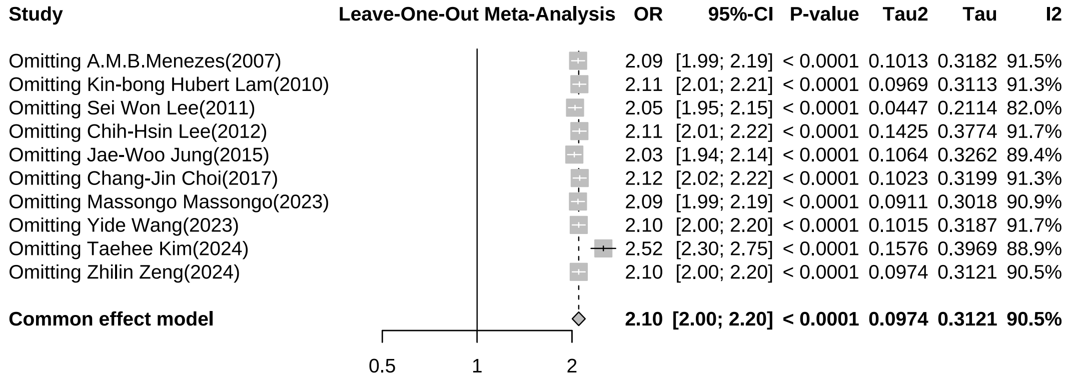

Figure S1. Sensitivity analysis for the association between tuberculosis and COPD risk using the leave-one-out method.

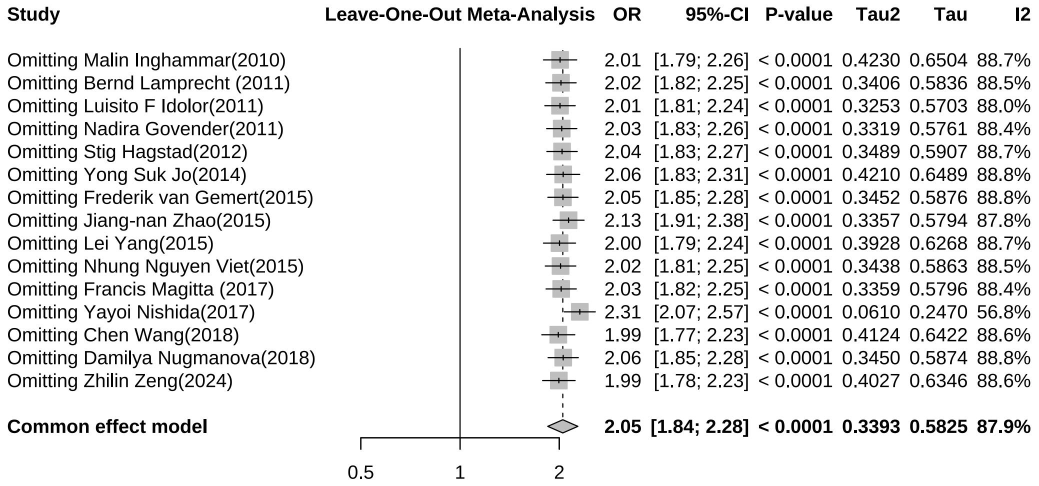

Figure S2. Sensitivity analysis for the association between COPD and tuberculosis risk

using the leave-one-out method.
